# Field modulation in Na-incorporated Cu(In,Ga)Se_2 _(CIGS) polycrystalline films influenced by alloy-hardening and pair-annihilation probabilities

**DOI:** 10.1186/1556-276X-6-581

**Published:** 2011-11-07

**Authors:** Yonkil Jeong, Chae-Woong Kim, Dong-Won Park, Seung Chul Jung, Jongjin Lee, Hee-Sang Shim

**Affiliations:** 1 Research Institute for Solar and Sustainable Energies (RISE), Gwangju Institute of Science and Technology (GIST), 261 Cheomdan-gwagiro, Buk-gu, Gwangju 500-712, South Korea; 2 Korea Institute of Industrial Technology, 1110-9 Oryong-dong, Buk-gu, Gwangju 500-757, South Korea

**Keywords:** Cu(In,Ga)Se_2_, solar cells, grain growth model, alloy hardening, pair-annihilation

## Abstract

The influence of Na on Cu(In,Ga)Se_2 _(CIGS) solar cells was investigated. A gradient profile of the Na in the CIGS absorber layer can induce an electric field modulation and significantly strengthen the back surface field effect. This field modulation originates from a grain growth model introduced by a combination of alloy-hardening and pair-annihilation probabilities, wherein the Cu supply and Na diffusion together screen the driving force of the grain boundary motion (GBM) by alloy hardening, which indicates a specific GBM pinning by Cu and Na. The pair annihilation between the ubiquitously evolving GBMs has a coincident probability with the alloy-hardening event.

**PACS: **88. 40. H-, 81. 10. Aj, 81. 40. Cd,

## Introduction

Thin film solar cells are promising candidates for power generation and other integrated photovoltaic applications, as part of an effort to develop new renewable energy technologies [1,2]. Specifically, chalcopyrite semiconductor systems, such as Cu(In,Ga)Se_2 _(CIGS), have attracted a great deal of interest as potential absorber materials for thin film solar cells. In recent years, the CIGS solar cells have demonstrated efficiencies of greater than 20% using three-stage co-evaporation methods [3]. One of the common methods for improving the performance of CIGS solar cells uses soda-lime glass (SLG) substrates, in which the amount of Na incorporated into the CIGS absorber layer is on the order of 0.1 at.% [4,5]. Several models have been proposed that explain the effect of Na on device performance. Wang et al. reported that the carrier concentration in the CIGS absorber layer increases due to a reduction in the amount of compensating (In,Ga)_Cu _defects due to the substitution with Na_Cu _[6-8]. In contrast, Herberholz et al. suggest that the existence range of α-CuInSe_2 _widens due to the incorporation of 0.1 at.% of Na, which suppresses the formation of the β-phase [9]. Rockett suggests that Na incorporation leads to an increase in the grain size and the lowest energy surfaces, such as Se-terminated (112) surfaces, due to an increase in the atomic mobility during CIGS growth and at grain boundaries [10,11]. Based on *ab initio *calculations, Persson and Zunger demonstrate that Na_Cu _defects or NaInSe_2 _phases at grain boundaries decrease the valence-band (VB) maximum due to a lack of Na d-electron states, which is similar to the case of (2*V*_Cu _+ In_Cu_) neutral defect complexes at grain boundaries [12,13]. However, the dominant phenomenon is an increase in the output voltage from the perspective of CIGS device physics, and an increase in the grain size from a crystallographic perspective. These increases are being recognized as far more acceptable explanations for the improvement in performance.

In this paper, we discuss how an increase in the fill factor is caused by the back surface field (BSF) effect from the perspective of CIGS device physics, and a structural change in the grain size from a crystallographic approach using a combination of alloy-hardening and pair-annihilation events.

## Experimental

### Fabrication of CIGS absorber film and device

CIGS solar cells were fabricated on Corning glass (CG) and SLG substrates. The basic properties, including the coefficient of thermal expansion (CTE), are summarized in Table 1 for the SLG and the CG [14] (http://www.abrisatechnologies.com). The polycrystalline CIGS films were deposited on Mo-coated SLG and CG using a typical three-stage co-evaporation process, as described in other studies [15,16]. In the first stage, indium (In), gallium (Ga), and selenium (Se) sources were evaporated at a growth temperature of 350°C to form a (In,Ga)_2_Se_3 _layer with a thickness of 1 μm. In the second stage, copper (Cu) and Se were evaporated and reacted with the (In,Ga)_2_Se_3 _layer at a temperature of 550°C to form the Cu-rich phase. In the third stage, In, Ga, and Se were evaporated to form the Cu-poor phase, while maintaining the substrate temperature. Then, CdS buffer and i-ZnO/Al:ZnO window layers were sequentially deposited. The cell area was defined as 0.49 cm^2 ^using a simple mechanical scribing tool.

**Table 1:** CTE and chemical composition of soda-lime glass and Corning glass substrates

Type	**CTE (×10**^**-6**^**/°C)**	Chemical contents (%)		
		**SiO**_**2**_	**Na**_**2**_**O**	Others
Soda-lime glass	8.4 (in the range of 25°C to approximately 513°C)	72.6	13.9	13.5
Corning glass	4.2 (in the range of 25°C to approximately 671°C)	69.0	1.0	30.0

### Characterization

The microstructure of the CIGS absorber layers was investigated using transmission electron microscopy (TEM, JEOL, Tokyo, Japan) operated at an acceleration voltage of 200 keV. The samples were prepared using a dual focused ion beam (FIB) system. Depth profiling of the chemical composition in both device structures was performed with a secondary ion mass spectrometer (SIMS, in a *Cameca IMS *4f system, CAMECA SAS, Gennevilliers Cedex, France) using an impact energy of 7.5 keV and a 200 nA O_2_^+ ^beam and detecting MCs + complexes (*M *= ^63^Cu, ^115^In, ^69 ^Ga, ^80^Se, ^23^Na, and^98^Mo). The solar cell efficiencies were measured and recorded using a Keithley 4300 source meter under 100-mW/cm^2 ^irradiation (Oriel^® ^Sol3A™, 450-W solar simulator equipped with an AM 1.5-G filter; Oriel Instruments, Irvine, CA, USA) and an incident-photon-to-electron conversion efficiency measurement system for the wavelength range of 300 to 1,200 nm (QEX7, PV Measurements Inc., Boulder, Colorado, USA).

## Results and discussion

### Microstructure and photovoltaic performances

**Figure 1:**
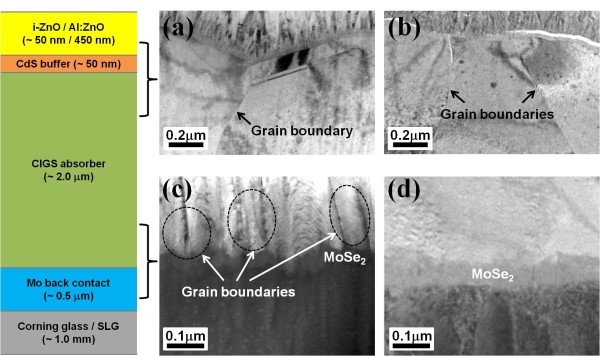
**XTEM images of each interface**. **(a) **the interface between the CdS buffer; **(b) **the CIGS absorber layers for the Na-restricted device and the Na-incorporated device; the interface between the CIGS absorber layers and the Mo back contact layers for **(c) **the Na-restricted device; **(d) **the Na-incorporated device.

Figure 1 shows the cross-sectional TEM (XTEM) images of the interfaces in the CIGS solar cells on the CG (Figure 1a,c) and SLG substrates (Figure 1b,d). The solar cells fabricated on the CG and the SLG substrates will be referred to as Na-restricted and Na-incorporated devices, respectively. The interfaces in Figure 1c,d have a quasi-ohmic MoSe_2 _layer formed by the inter-diffusion of Se and Mo atoms [17,18]. In Figure 1a,b, the grain configurations, such as the size and crystallographic orientation, seem to be similar to one another, while the bottom regions of the CIGS absorber layers in Figure 1c,d clearly show different features. The interface configuration in Figure 1c might be that of the elongated grain boundary motion (GBM) between the CIGS polycrystalline grain blocks, moving toward the Mo surface.

**Figure 2:**
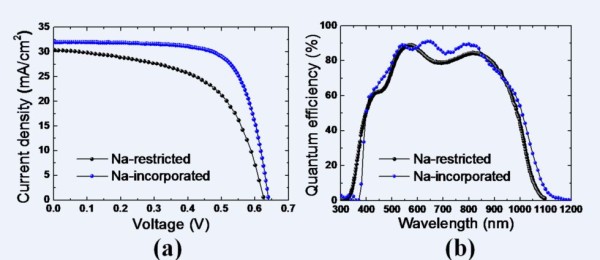
**J-V and EQE curves**. **(a) **Illuminated J-V and **(b) **EQE curves of Na-restricted and Na-incorporated devices.

Figure 2 shows the illuminated J-V and external quantum efficiency (EQE) curves of the Na-restricted and Na-incorporated devices. The open-circuit voltage (*V*_oc_), short-circuit current density (*J*_sc_), fill factor (FF), and photo-conversion efficiency (Eff) are summarized in Table 2. The EQE curves exhibit similar absorption band-edges, which represent a very small difference in the output voltage, as shown in Figure 2b. From the EQE in the wavelength range of 400 nm to approximately 550 nm and 600 nm to approximately 850 nm in Figure 2b, the slightly higher quantum efficiency of the Na-incorporated device leads an improvement in the *J*_sc_. However, the main source of efficiency improvement, which increases significantly from 10.9% to 14.6%, is caused by the enhancement in the FF of greater than 10%. This result could be attributed to the BSF induced at the bottom region of the CIGS absorber layer. The BSF effect might not be from the energy band-gap tuning by the In and Ga profile modulation but instead from the energy-level pinning caused by a structural change in the Na-incorporated CIGS absorber layer.

**Table 2:** Parameters obtained from illuminated J-V curves.

Device	***V***_**oc **_**(V)**	***J***_**sc **_**(mA cm**^**-2**^**)**	FF (%)	Eff (%)
Na restricted	0.626	30.4	57.1	10.9
Na incorporated	0.641	32.0	71.1	14.6

### Compositional profile

**Figure 3:**
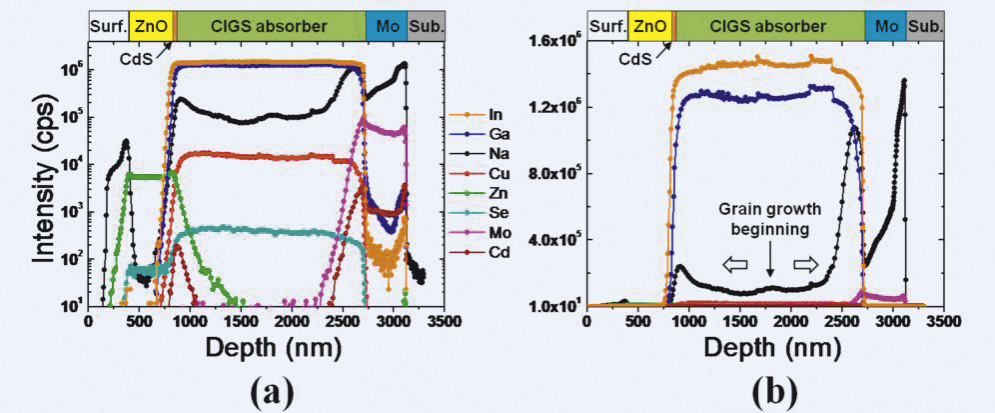
**SIMS depth profile**. Plotted on (a) a logarithm scale and **(b) **a linear scale for the Na-incorporated devices.

Figure 3 shows the SIMS profile plotted on a logarithm and linear scale for the Na-incorporated device as a function of the sputter-etch time (the SIMS profile of the Na-restricted device is not shown in here). The Na is distributed from the substrate to the surface: the Na intensity is higher than that of Cu and Se in the CIGS absorber layer, whereas it is lower than that of In and Ga (Figure 3a). In particular, the gradient of the Na profile changes abruptly at the interface between CIGS and Mo. This finding implies two possible diffusion mechanisms of elemental Na. First, the Na continuously diffuses through the Mo from the SLG during the CIGS grain growth, in which Na diffusion is gradually restricted by the compact CIGS grain blocks, and consequently, Na accumulation is more prominent in the bottom region of the CIGS absorber layer. Second, the Na diffusion should already be in progress during the deposition of the (In,Ga)_2_Se_3 _precursor film, and in the beginning of the CIGS grain growth, Na should begin to spread out upward and downward, as shown in Figure 3b. At the same time, the continuous Na diffusion from the substrate results in a prominent Na profile in the bottom region. In practice, such a Na profile could improve the device performance, as reported in the literature [19]. As far as the Na diffusion mechanism is concerned, the second mechanism seems to be a far more acceptable explanation. Herein, we suggest a model describing a structural change in the CIGS polycrystalline film.

### Grain growth model

**Figure 4:**
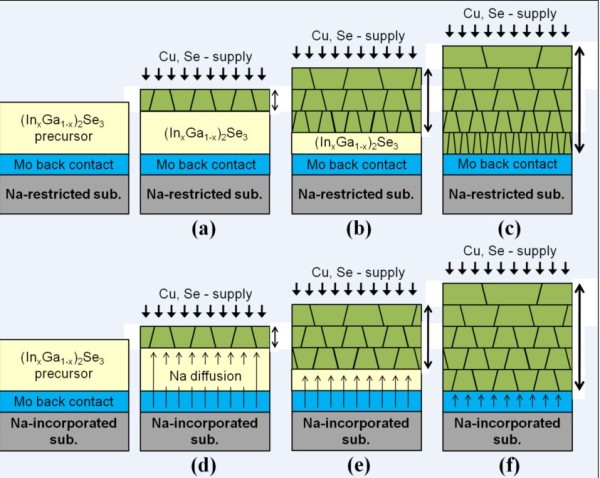
**The mechanism of the crystal growth model for CIGS films**. In **(a,b,c) **Na-restricted and **(d,e,f) **Na-incorporated devices.

Figure 4 provides a detailed schematic of the structural change that occurs during the growth process of the CIGS film for the Na-restricted device (Figure a,b,c) and the Na-incorporated device (Figure d,e,f). The (In*_x_*Ga_1-*x*_)_2_Se_3 _precursor film is formed in the first deposition stage of the CIGS film. For the second stage in which Cu and Se are introduced, the grain growth of the CIGS starts from the surface of the (In_x_Ga_1-x_)_2_Se_3 _film (Figure 4a,d). The grain growth and volume expansion of the CIGS crystal would progress simultaneously upward and downward, leading the GBM. The upward grain growth from the initially formed surface grain results from alloy-hardening and pair-annihilation events in both devices [20-22]. The constant Cu supply to the surface leads to a lower activation energy for the GBM [23,24] because the supplied Cu atoms induce an alloy hardening in the CIGS film and make the upward propagation of the grain boundary more energetically unfavorable. In addition, Cu could act as a driving force screen for the GBM, which indicates that the GBM is partially pinned by the Cu supply. Subsequently, the grain boundaries come together in the same growth direction and are eventually pair-annihilated. The grain size at the top region of the CIGS films, as shown in Figure 1a,b, can be explained by the upward structural evolution. However, the downward structural evolution occurs in a different manner in the Na-restricted and the Na-incorporated devices. In the case of the Na-restricted film, the downward grain growth is accompanied by a volume expansion that weakens the grain size effect because the Cu diffusion is gradually restricted due to the compactly preformed CIGS crystal grains. Finally, the polycrystalline CIGS film is formed as shown in Figure 4c, which is attributed to the structural configuration of the bottom region in Figure 1c. In the Na-incorporated film, the downward grain growth should be the same as that of the Na-restricted device. However, there is sufficient Na diffusion from the SLG such that the Cu vacancies are compensated due to the limited diffusion rate of Cu and it maintains grain growth by alloy hardening. Such a structural feature of the bottom region (Figure 4f) is in good agreement with the TEM image, as shown in Figure 1d. It should be noted that the Na diffusion from the SLG occurs during the second stage due to a higher CTE of the SLG substrate, while the Corning glass substrate restricts the Na diffusion due to its lower CTE and lower Na content. From the SIMS profile, we observe that the Na is prominently located at the interface between the CIGS absorber and the Mo back contact, in which the Na might occupy the Cu vacancies or replace the Cu atoms and form Na(In,Ga)Se_2 _phases, which produces a decrease in the valence-band maximum at the grain boundaries [12,13]. In other words, the electric field is modulated because the valence-band maximum approaches the Fermi-energy level. Specifically, the prominent Na profile in the bottom region of the CIGS absorber layer is likely to cause energy-level pinning, which could strengthen the BSF.

## Conclusions

We fabricated Na-restricted and Na-incorporated CIGS solar cells to investigate the influence of Na on the device performances. The enhancement in the output voltage and photocurrent density for the Na-incorporated device was negligible, while the FF revealed a remarkable increase. This finding could be attributed to the strengthening of the BSF by the energy-level pinning in the bottom region of the CIGS absorber layer. The energy-level pinning originates from the proposed grain growth model wherein the Cu supply and the Na diffusion both contribute to the grain growth by a combination of alloy-hardening and pair-annihilation events that occur between grain boundaries.

## Abbreviations

CIGS: Cu(In,Ga)Se_2_; GBM: grain boundary motion; SLG: soda-lime glass; CG: Corning glass; VB: valence band; BSF: back surface field; CTE: coefficient of thermal expansion; TEM: transmission electron microscopy; FIB: focused ion beam; SIMS: secondary ion mass spectrometer; XTEM: cross-sectional TEM; EQE: external quantum efficiency; *V*_oc_: open-circuit voltage; *J*_sc_: short-circuit current; FF: fill factor; Eff: photo-conversion efficiency.

## Competing interests

The authors declare that they have no competing interests.

## Authors' contributions

YKJ, CWK and HSS designed and drafted the study. CWK fabricated the CIGS absorber films and devices using three-stage co-evaporation technique. YKJ and HSS carried out the characterization of the CIGS devices. DWP and JJL participated in the establishment of the grain growth mechanism for CIGS absorber film during the three-stage evaporation. All authors read and approved the final manuscript.
